# Multimodal chest surface motion data for respiratory and cardiovascular monitoring applications

**DOI:** 10.1038/sdata.2017.52

**Published:** 2017-04-25

**Authors:** Ghufran Shafiq, Kalyana Chakravarthy Veluvolu

**Affiliations:** 1 School of Electronics Engineering, Kyungpook National University, Daegu 702–701, South Korea

**Keywords:** Radiotherapy, Diagnosis, Biomedical engineering

## Abstract

Chest surface motion is of significant importance as it contains information of respiratory and cardiac systems together with the complex coupling between these two systems. Chest surface motion is not only critical in radiotherapy, but also useful in personalized systems for continuous cardiorespiratory monitoring. In this dataset, a multimodal setup is employed to simultaneously acquire cardiorespiratory signals. These signals include high-density trunk surface motion (from 16 distinct locations) with VICON motion capture system, nasal breathing from a thermal sensor, respiratory effort from a strain belt and electrocardiogram in lead-II configuration. This dataset contains 72 trials recorded from 11 participants with a cumulative duration of approximately 215 min under various conditions such as normal breathing, breath-hold, irregular breathing and post-exercise recovery. The presented dataset is not only useful for evaluating prediction algorithms for radiotherapy applications, but can also be employed for the development of techniques to evaluate the cardio-mechanics and hemodynamic parameters of chest surface motion.

## Background & Summary

Chest surface motion can provide vital signs: respiration and palpitations on the chest due to beating heart. These two vital signs are important and find their use in cardiac and respiration related applications. For example, respiratory signals aid in effective radiotherapy for treating lung-tumours, while the vibrations due to beating heart are useful in long-term cardiac health monitoring systems.

Radiotherapy is an alternative for treating tumours where surgical treatment is either undesired or not feasible^1,2^. In the process, high dosage of radiation is focused on the tumour affected tissues while avoiding the dosage to healthy tissues. However, one of the challenging problems in robotic radiotherapy treatment is the movement of the tumour due to body motion. The problem worsens especially in lung tumour, where the movement of the tumour due to respiration is significant. This motion poses a risk of exposing healthy tissues to the radiation. Chest surface movement of the patient is generally correlated to tumour movement and therefore can be proven as a good predictor of the tumour movement^3^. However, there exists a delay between actual tumour movement and the estimated movement from correlation model due to inherent mechanical limitations, image acquisition and processing time^4^. This delay results in erroneous positioning of the radiation beam on the tumour and can be avoided if the body (respiratory) motion can be estimated in advance for some duration^3–5^. Datasets are publicly available but they only have three markers on the body to represent the whole body motion. In our set-up, we have placed 16 markers in a grid configuration that effectively covers the trunk of the patient such that the prediction algorithms can be tested extensively. Further, methods for respiratory rate variability analysis similar to^6–8^ can also be developed or tested with this dataset.

Chest surface motion also contains vibrations due to beating heart that can be useful for developing personalised long-term cardiac health monitoring systems. Such systems aim to provide early warning systems for heart diseases, where symptoms appear sporadically or are unnoticeable to patients^9,10,11^. Chest vibrations can be recorded with cost-effective, small sized, light weight and multi-purpose inertial sensors which can be used simultaneously for other applications such as fall detection^12^, sleep analysis^13^ and energy expenditure estimation in physical activity^14^. The utilisation of chest vibration signal for automatic classification of cardiac arrhythmias^10^ and early stage haemorrhage detection^14^ has been successfully demonstrated in the literature. However, to the best of author’s knowledge, these works are based on single sensor. Hence, the chest vibration signal should be recorded under controlled laboratory environment to minimize motion artefacts. Otherwise, if these artefacts cannot be removed, the motion corrupted segment of the signal is rendered useless and should be discarded^9^. Employing multiple sensors in combination with appropriate signal processing techniques can cancel the artefacts from sources such as respiration, body motion or other involuntary activities (coughing or sneezing etc.). In our previous work^15^, a subset of this dataset has been employed in an effort to develop approaches for separation of cardiac motion from chest surface motion. These approaches are based on the framework of Independent Component Analysis with reference (ICA-R) to separate cardiac motion from the chest surface motion acquired from multiple locations on the chest. The dataset is openly released for the first time with this data descriptor.

In these experiments, motion from sixteen different locations was collected, forming a grid over the subject’s trunk. In radiotherapy applications, higher number of locations will result in an improved estimate of trunk motion above the tumour without requiring any approximate modelling. Therefore, the actual tumour motion can be identified with an improved accuracy. For cardiovascular monitoring applications, this dataset can help researchers to investigate the optimal locations and optimal number of motion sensors on the body to ensure effective cancellation of respiration and other artefacts. However, for investigation purposes, it is difficult to employ and synchronise a large number of accelerometers. Therefore, we used an Infrared (IR) optical motion tracking system to record trunk motion in three dimensions. Techniques can be developed to optimise both the number of sensors and their locations for effective removal of the artefacts. This will eventually facilitate the replacement of optical markers with fewer inertial sensors.

## Methods

### Participants

Eleven male participants 25.36±2.93 years old with BMI 22.39±2.33 kg m^−2^ responded to the in-campus announcement and volunteered for this study. All participants were healthy and reported no previous record of any cardiac or pulmonary disease. The subjects were clearly briefed about the nature of the experiment. Participants gave their informed consent for participating in the experiment as well as for allowing the data to be shared publicly after de-identification. The study was approved by the institutional review board of Kyungpook National University and was conducted according to the principles expressed in the declaration of Helsinki.

### Procedures

With informed consent, the subjects were requested to provide their personal information such as age, weight, height, trunk measurements, smoking habits, alcohol consumption in past 24 h and sleep duration for the last night. This information is presented in Table 1 for all the 11 subjects. Subjects S1–S9 participated in our earlier related work^15^, while data from S10–S11 was acquired at a later stage.

The experiment consisted of simultaneous data acquisition of: (i) Trunk surface motion from VICON Infrared (IR) Motion capture system (VICON Motion Systems Ltd., UK), and (ii) reference physiological signals such as electrical heart activity, chest motion and respiratory airflow signal from BIOPAC MP36 (BIOPAC Systems Inc., USA). The subsequent methods explained here are elaborated versions of the descriptions provided in our related work^15^. However, the previous work^15^ utilised only the surface trunk motion from VICON, while ECG was used for validation. Here, signals from respiratory strain belt and nasal thermistor (airflow) are also provided.

### Surface trunk motion (VICON)

**Figure 1:**
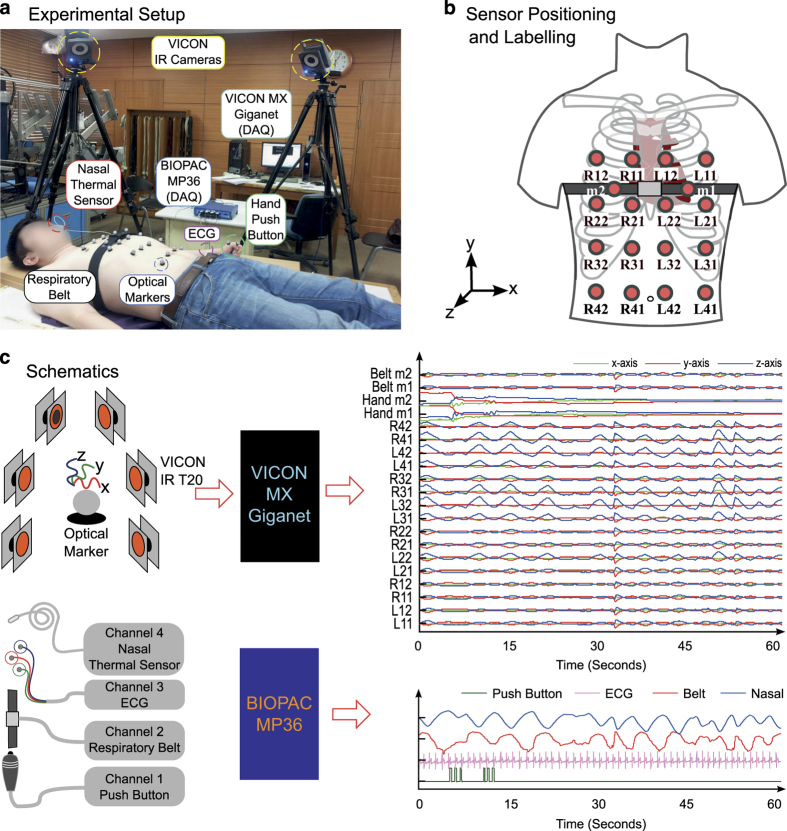
Experimental Setup and Data acquisition. (**a**) Experimental Setup with participant in supine posture, (**b**) Positioning and Labelling of optical markers for VICON motion capture, and placement of respiratory effort belt, (**c**) Example of all the acquired signals from two systems for a free breathing trial. Fig. 1a,b are taken from our previous work^15^.

The trunk surface motion of the subject was recorded using six VICON IR cameras placed in a nearly circular pattern around the subject lying supine on the table as shown in Fig. 1a. Each of these cameras is equipped with an IR strobe. The IR light emitted from the strobe is reflected by retro-reflective optical markers back to the camera. All six VICON T20 IR cameras were connected to VICON MX Giganet, which was eventually connected to a personal computer (PC). The PC was running VICON NEXUS software, which recorded the raw data from all cameras and reconstructed the three-dimensional motion for each of the optical markers. Sixteen such markers are placed on the anterior of the human trunk in a 4×4 grid as shown in Fig. 1b. A similar grid pattern is also employed in ref. 16 to facilitate modelling and prediction of temporal changes in the chest wall surface. The grid is roughly equally distributed from left mid clavicle line to right mid clavicle line and upper pectoral muscles to abdomen (navel). Placement of markers was done to capture the motion of the full trunk. Efforts were made to adjust the optical markers to cover the point of maximal impulse (PMI) and lower sternum to ensure maximal and clean heart-related chest movement. Markers are labelled as L_ij_ and R_ij_, where i={1,2,3,4} is the row in the grid, whereas j={1,2} is the column in each left and right side of the grid. The circular configuration of the cameras was adjusted such that each optical marker was visible to at least three cameras at any given time for reliable 3D reconstruction. Further, the strobe intensities and light detection threshold of each camera were set to ensure the visibility of only optical markers and also to eliminate the stray reflections from undesired objects. Care was also taken to avoid direct exposure of the IR strobe of one camera to another. Sampling rate for the VICON system was set to 100 frames per second.

### Reference physiological signals (BIOPAC)

Along with the surface trunk motion, simultaneous recording of three reference signals was performed using a BIOPAC MP36 data acquisition unit connected to a PC as shown in Fig. 1c. The three reference signals are: (i) Electrocardiogram (ECG), (ii) Chest motion from the respiratory belt (strain gauge), and (iii) respiratory airflow signals from the nasal thermal sensor. The sampling frequency of the BIOPAC MP36 unit was set to 100 Hz.

#### ECG

A single channel BIOPAC electrode lead set (SS2LA) was connected to channel 3 of BIOPAC MP36. The electrode lead set was attached to three disposable vinyl electrodes that were placed on the right anterior wrist and medial surface of each leg (just above the ankle) of the subject. In this way, Lead II configuration was established. To ensure hygienic practice, only one set of electrodes were used per subject and they were disposed of after usage.

#### Chest motion

A pneumograph transducer or nylon respiratory belt (SS5LB) was attached to subject’s chest located approximately just above the midsternal line as shown in Fig. 1b. The compression and expansion of the chest during breathing changes the tension in the respiratory belt. This change in tension is converted into voltage by the transducer. Care was taken in adjusting the tension of the respiratory belt to ensure that the belt was slightly tight at the point of maximal chest compression (maximal expiration). The output of SS5LB was connected to channel 2 of the BIOPAC MP36.

#### Airflow

A Fast Response Thermistor (SS6L) was connected to channel 4 of the BIOPAC MP36, and was placed just below one of the subject’s nostrils. During normal breathing cycle, exhaled air is warmer than the cold inhaled air. Therefore, the fast response thermistor can measure the airflow in and out of the nostrils.

### Experiment protocol and trials

**Figure 2:**
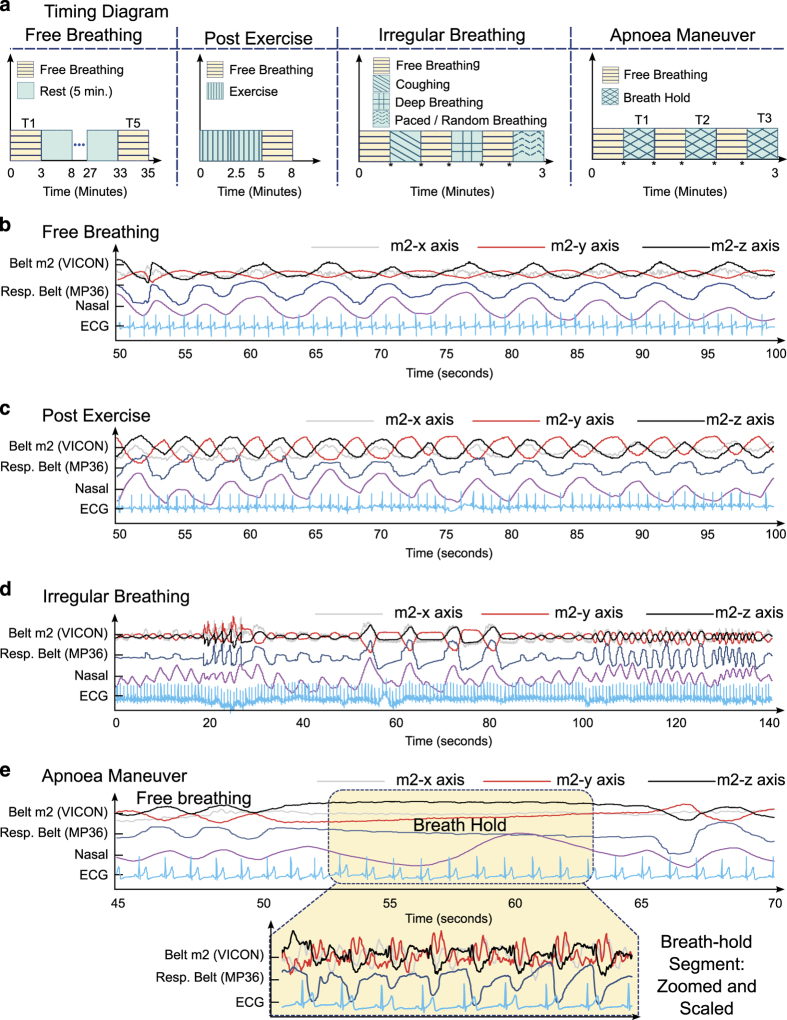
Data acquisition protocol and sessions. (**a**) Timing diagram for the all the sessions, indicating timeline of the recording sessions, and illustration of example signals from (**b**) Free breathing, (**c**) Post Exercise, (**d**) Irregular Breathing and (**e**) Apnoea Manoeuvre sessions.

The subjects were asked to lie down on a table in supine position and breathe freely (unless in the case of pre-defined manoeuvres). The data acquisition session was divided into four sessions: (i) free breathing, (ii) breath hold, (iii) irregular breathing, and (iv) post-exercise. The timing diagram for each session is illustrated in Fig. 2a. The distribution of trials per session for all the subjects is shown in Table 2.

#### Free breathing

In this session, the subjects were asked to breathe normally with no special manoeuvres. The subjects were asked to remain calm, avoid unnecessary body motion and talking during the trial recording. The recording duration for one trial was 180 s. 52 such trials were recorded from 11 subjects. Four of the subjects volunteered to participate in three trials, one subject participated in 8 trials, one subject participated in 4 trials and 5 subjects participated in 5 trials each. For illustration, a segment from a free breathing trial is shown in Fig. 2b.

#### Post-exercise

In this session, the subjects were asked to perform a moderate exercise (jumping jacks) for approximately 3–5 min and then lie down on the table. After the subject lies down on the table, the trial is recorded for approximately 3 min in which the subject breathes to recover from the exercise. Five subjects participated in this session for which one trial was recorded per subject. For illustration, signals recorded after exercise are shown in Fig. 2c. Increase in both the heart rate and respiration rate are observed as compared to the free breathing trial in Fig. 2b.

#### Irregular breathing

In this session, the subjects were asked to perform irregular breathing manoeuvres in a single 3-minute trial. The manoeuvres consisted of paced breathing, deep breathing, coughing and erratic breathing. The duration of these manoeuvres was left on the subjects’ discretion to ensure their comfort. During trial recording, the subjects were asked to remain calm, avoid unnecessary movements and talking. Six subjects participated in this session and one 3-minute trial per subject was recorded. For illustration, a segment of signals recorded during an irregular breathing trial is shown in Fig. 2d.

#### Apnoea manoeuvre

In this session, the subjects were asked to perform a controlled apnoea manoeuvre (holding breath) for a comfortable duration. During the trial recording, subjects were asked to remain calm, avoid unnecessary movements and talking. The recording started by free breathing for few seconds, followed by breath hold for few seconds, followed by recovery and again followed by breath hold. Therefore, a single 3-minute trial per subject contains multiple instances of breath hold segments. The number and duration of these segments were left at the subject’s discretion to ensure their comfort. Two such trials were recorded for one subject, while one trial each was recorded for the remaining ten subjects. For illustration, a segment of signals recorded in an apnoea manoeuvre trial is shown in Fig. 2(e). The highlighted portion indicates the duration where subject held breath. Alignment of the ECG R-peaks with the chest motion signals (from respiratory belt and z-axis of VICON m2 marker) can be observed in the zoomed and scaled highlighted segment.

### Code availability

All the custom codes used in the technical validation of this study were created in MATLAB R2015a and are freely available at https://github.com/gshafiq/SData_CDM.

## Data Records

Data records presented in this section and accompanying detailed description file (README) are available online from figshare (Data Citation 1). Data from each trial are stored in standard MAT files (MATLAB), which are converted from CSV format using MATLAB import tool.

For each session (free breathing, breath hold, irregular breathing and post-exercise), data are stored in separate folders. The files are de-identified in the format conversion process, where subjects’ names are replaced by subject ID. Each folder contains trials with names ‘<Trial Type>_Txx’, where xx is the incremental integer. Table 3 shows the assignment of the trial name for each subject for all the sessions. For instance, in Free breathing archive, Free_T1.mat, Free_T2.mat and Free_T3.mat belongs to subject S1.

Each.mat file contains three matrices named as vicon_s, mp36_s and ofst. There are 60 rows in vicon_s matrix. Each (3i+1)th row represents x-axis, (3i+2)th row represents y-axis and (3i+3)th row represents z-axis of motion of the optical markers for i=0,1,…,19. The labelling for optical markers and their corresponding rows in vicon_s.mat are given in Table 4.

Data from BIOPAC mp36 are stored in mp36_s matrix that contains four rows. Each row in mp36_s corresponds to signals from: (i) Hand switch, (ii) Respiratory belt, (iii) ECG and (iv) Nasal Thermal sensor. The ‘ofst’ variable consists of the offset value between VICON and BIOPAC mp36 unit during recording, which is already corrected in the.mat files.

For breath hold trials, two additional vectors ‘s_pos’ and ‘e_pos’ are defined which contain starting and ending positions of the breath hold attempts in a given trial. For instance, s_pos(1) and e_pos(1) correspond to starting point and ending point of the first breath hold attempt for the given trial.

## Technical Validation

### Synchronization between BIOPAC and VICON

The BIOPAC Data acquisition hardware (MP36) and the VICON motion capture system were connected to two separate computers for operation and storage. For synchronisation, an SS10L push button hand switch was connected to the channel 1 of the MP36 unit (as shown in Fig. 1c). The subjects were asked to grip the switch in hand with thumb resting on the push button. Two additional markers were placed on the subjects’ thumb (nail and metacarpal bone). After starting the recording on both the computers, the subjects were asked to press the push button with thumb three times. Upon push of the button, a 5 mV signal is generated. The signal returns to 0 V when the button is released. Simultaneously, downward movement of the thumb is recorded by the VICON system. For coarse synchronisation, time offset between push button signal from the MP36 system and the corresponding downward thumb motion from the VICON system is determined and the offset is corrected. The subjects were instructed to press the push button again three times at the end of recording for reliability. During breath hold and irregular breathing sessions, the subjects were instructed to press the push button once before and after the performed manoeuvre. These additional presses allow easier segmentation of the manoeuvre in post-processing stage.

### Comparison of respiratory signal modalities

The similarity between respiratory signals from the nasal thermal sensor, VICON motion capture and strain belt was demonstrated with wavelet phase coherence. For this purpose, the coherence was identified for three pairs of signals: (i) VICON and strain belt, (ii) VICON and nasal thermal sensor, and (iii) strain belt and nasal thermal sensor. The wavelet phase coherence for two signals *x*_1_(*t*) and *x*_2_(*t*) in each pair is identified as^17,18^:

(1)C∅(ω)=1N(∑n=1Nsin[∅(tn)])2+(∑n=1Ncos[∅(tn)])2

with

(2)sin[∅(tn)]=b1,w,na2,w,n−a1,w,nb2,w,na1,w,nb1,w,na2,w,nb2,w,n

and

(3)cos[∅(tn)]=a1,w,na2,w,n+b1,w,nb2,w,na1,w,nb1,w,na2,w,nb2,w,n

where *a*_*k,w,n*_ and *b*_*k,w,n*_ are real and imaginary coefficients of the wavelet transform *X*_*k*_(*w*_*n*_, *t*_*n*_) of the signal *x*_*k*_(*t*), {*k*=1,2} such that *X*_*k*_(*w*_*n*_, *t*_*n*_)=*a*_*k,w,n*_+*i b*_*k,w,n*_. The phase coherence ranges from 0 (mutually unrelated oscillations) to 1 (mutually related oscillations) at a given frequency.

The scales were chosen to analyse the signals in the frequency range of 0.05 and 50 Hz. Further, 500 scale points were logarithmically distributed to increase the frequency resolution in lower frequencies that are dominated by the respiratory signal and its harmonics. Equation (1) provides the wavelet phase coherence for whole frequency range under analysis. However, a scalar measure is required to provide one value for a specific pair of signals in a given trial. This measure will facilitate the subsequent statistical analysis of all the trials in a session. For this purpose, maximum phase coherence in a selected band (MPCB) was computed for all the trials. The selected band of [0.1, 0.6] Hz represents respiratory dominant frequency band. A higher frequency range was selected to compensate for higher breathing rates in the irregular breathing and post-exercise trials.

**Figure 3:**
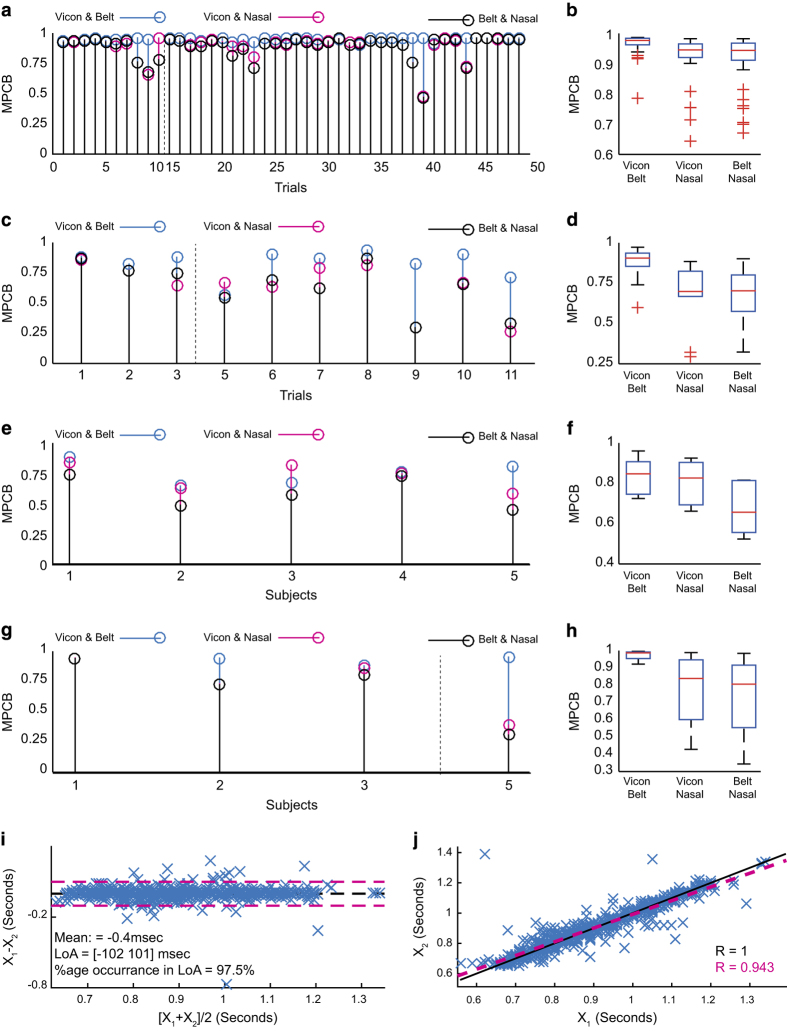
Technical Validation. Maximum Phase Coherence in selected Band (MPCB) between the signals from VICON (marker M2), respiratory effort belt and Nasal thermal sensor (airflow) for free breathing (**a**,**b**), apnoea manoeuvre (**c**,**d**), irregular breathing (**e**,**f**) and post exercise trials (**g**,**h**). Bland-Altman Analysis (**i**) and Scatter plot (**j**) of R-R intervals obtained from VICON (marker L22-zaxis) and ECG apnoea manoeuvre attempts in all trials.

Prior to identification of the MPCB, drift and high-frequency noise were removed from each signal by applying a zero-phase bandpass filter from 0.08 to 10 Hz. Marker M2 (on belt) was selected from the VICON system for coherence analysis. Trial-wise MPCB between the three pairs of signals for all the trials in free breathing, apnoea manoeuvre, irregular breathing and post-exercise conditions are shown in Fig. 3a,c,e,g respectively. Further, median and interquartile ranges of the MPCB are illustrated by box plots in Fig. 3b,d,f,h. Few trials from free-breathing (trials 11–14 and trials 49–51), apnoea manoeuvre (trial 4) and post-exercise (trial 4) were not included in the analysis. In these trials, the belt was not tightened enough due to an indication of discomfort from the subjects. Loosening of the belt resulted in either no signal or highly distorted or unreliable respiratory signal.

For simplicity, MPCB between the three pairs i.e., (i) VICON and Belt, (ii) VICON and Nasal and (iii) Belt and Nasal are represented by MPCB_V-B_, MPCB_V-N_ and MPCB_B-N_ respectively. As illustrated in Fig. 3a, out of 44 free-breathing trials, MPCB_V-B_, MPCB_V-N_ and MPCB_B-N_ are observed greater than 0.9 in 43, 38 and 35 trials respectively. From Fig. 3b, it is observed that the median of MPCB_V-B_ is the highest, while the medians of MPCB_V-N_ and MPCB_B-N_ are similar. However, the inter-quartile range of the MPCB_V-N_ is smaller as compared to MPCB_B-N_. The MPCB for the three pairs in apnoea manoeuvre trials is shown in Fig. 3c,d. Out of 10 trials, MPCB_V-B_, MPCB_V-N_ and MPCB_B-N_ are greater than 0.6 in 9, 8 and 7 trials respectively. It is observed from Fig. 3e,f that out of 5 trials, MPCB_V-B_, MPCB_V-N_ and MPCB_B-N_ for irregular breathing trials are greater than 0.7 in 4, 3 and 2trials respectively. The MPCB for post-exercise trials is shown in Fig. 3g,h. It is observed that out of 4 trials, MPCB_V-B_, MPCB_V-N_ and MPCB_B-N_ are greater than 0.7 in 4, 3 and 3 trials respectively.

Overall, the MPCB between the three pairs of modalities is higher than 0.5 for all the recording conditions, which implies that the signals are related. The medians of MPCB_V-N_ and MPCB_B-N_ are similar in all conditions except the irregular breathing session. The MPCB between VICON and Belt (MPCB_V-B_) has the greatest median value and the smallest inter-quartile range in all recording conditions, implying that the similarity of these two signals are higher as compared to the remaining two pairs. Further, it is observed that in most of the trials, MPCB_V-B_ is highest, followed by MPCB_V-N_ and MPCB_B-N_ in all recording conditions. This trend is expected as the signals from VICON and Belt are similar in nature as both measure mechanical chest motion, while the signals from Nasal sensor are based on airflow temperature.

### Comparison of cardiac signals

The applicability of chest motion in cardiac applications is validated by demonstrating the similarity between heart beat intervals and R-R intervals obtained from chest motion and ECG respectively. The z-axis of marker L22 in the VICON system was selected as the chest motion signal for this analysis. Furthermore, breath hold signals were chosen as the absence of respiration simplifies further analysis. However, several respiration removal methods have been developed that are based on polynomial curve fitting^19^, adaptive cancellation of respiratory harmonics^20^, non-linear separation scheme^21^ and constrained Independent Component Analysis (cICA)^15^ etc.

Every breath-hold trial was segmented such that each of the segments contained an apnoea manoeuvre attempt. For every segment, the R-R intervals were calculated by detecting the R-peaks in the ECG signal and then identifying time difference between the consecutive peaks. To facilitate the detection of R-peaks, baseline wander was estimated and removed from the ECG signal by a moving average filter with window length of 20 samples (0.2 s). Similarly, the baseline wander in the chest motion signal was removed by a moving average filter with window length of 80 samples (0.8 s) to facilitate the peak detection. Further, due to complex nature of the chest motion waveform, a sliding correlation approach was employed for peak detection, which is similar to template matching method presented in ref. 22. A template was selected from the chest motion signal, which started at 3-second mark and ended at 5-second mark in each segment. There was no specific preference for the location (and therefore phase) of the selected template. For every segment, a time-lag cross-correlation function between the template and the chest motion was computed. Peaks in the cross-correlation function were detected and the time interval between the consecutive peaks was calculated. This process was repeated for 32 breath-hold attempts in total. However, two of these attempts (attempt 1 from trial 1 and attempt 2 from trial 2) were excluded from the subsequent analysis due to undesired artefacts that affected the peak detection.

The peak-peak time-intervals in chest motion signals (VICON) and the R-R intervals in ECG signals are represented by X_1_ and X_2_ as shown in Fig. 3i,j. The Bland-Altman analysis^23^ is performed on the time-intervals X_1_ and X_2_ as shown in Fig. 3i. The mean difference between the intervals is indicated by a black solid line, while the red dashed lines show the limits of agreement (LoA). The LoA is identified as Mean (X_1_−X_2_) ±1.96STD (X_1_−X_2_), where STD is the standard deviation. It is observed that the difference in the intervals from ECG and the chest motion signals does not have any growing or decaying trend over the entire range of intervals. Further, the mean difference between the intervals is quite negligible (−0.4 ms) and the limits of agreement are from −102 ms to 101 ms, which contains 97% of the data points. The scatter plot in Fig. 3j shows the similarity between the peak-peak time intervals from the chest motion signal and R-R intervals from the ECG signals. The red dashed line represents the regression line for the heart beat intervals, whereas the black line represents an ideal regression line. It can be observed that the scattering is quite small with a high Pearson’s correlation coefficient (R=0.943).

## Usage Notes

The complete dataset is online available at figshare (Data Citation 1). All records are stored in MATLAB compatible.mat files and are categorised based on recording conditions (normal breathing, breath-hold etc.). However, these files can be converted into.csv or other applicable formats for usage with other programming tools.

## Additional Information

**How to cite this article:** Shafiq, G. and Veluvolu, K. C. Multimodal chest surface motion data for respiratory and cardiovascular monitoring applications. *Sci. Data* 4:170052 doi: 10.1038/sdata.2017.52 (2017).

**Publisher’s note:** Springer Nature remains neutral with regard to jurisdictional claims in published maps and institutional affiliations.

## Supplementary Material



## Figures and Tables

**Table 1:** Personal Information of all the subjects.

**Subject**	**Age (Years)**	**Weight (kg)**	**Height (cm)**	**BMI (kg m^−2^)**	**Trunk Length (cm)**	**Trunk Width (cm)**	**Smoking (Yes/No)**	**Sleep duration for Last Night (Hours)**
S1	25	56	168	19.8	33	22	Yes	8
S2	24	62	173	20.7	34	22	No	8
S3	26	63	168	22.3	35	36	Yes	8
S4	22	65	178	20.5	32	22	Yes	6
S5	28	74	178	23.4	32	20	No	5
S6	22	61	168	21.6	28	22	No	6.5
S7	32	53	170	18.3	32	24	No	7
S8	23	68	158	27.2	28	20	No	7
S9	28	70	175	22.9	33	23	Yes	4
S10	23	75	172	25.4	34	24	No	4
S11	26	75	176	24.2	34	30	Yes	8
Mean±STD	25.36±2.93	65.63±6.49	171.27±5.45	22.39±2.33	32.27±2.21	24.09±4.51	N/A	6.5±1.42

**Table 2:** Distribution of trials in each session for the subjects.

**Subject**	**Free Breathing**	**Breath Hold**	**Irregular Breathing**	**Post-Exercise**
S1	3	2*	—	—
S2	3	1	—	—
S3	8* (4-4*)	1	1	1
S4	5	1	1	1
S5	5	1	—	—
S6	5	1	1	1
S7	4	1	1	1
S8	5	—	1	1
S9	5	1	—	—
S10	3	1	—	—
S11	3*	1	—	—
Total	51	11	5	5

**Table 3:** Trial numbers for each subject.

**Subject ID**	**Free Breathing (Trial)**	**Breath Hold (Trial)**	**Irregular Breathing (Trial)**	**Post-Exercise (Trial)**
S1	1–3	1–2	—	—
S2	4–6	3	—	—
S3	7–14	4	1	1
S4	15–19	5	2	2
S5	20–24	6	—	—
S6	25–29	7	3	3
S7	30–33	8	4	4
S8	34–38	—	5	5
S9	39–43	9	—	—
S10	44–48	10	—	—
S11	49–51	11	—	—
Total	51	11	5	5

**Table 4:** Optical Marker Label and the corresponding rows in matrix ‘vicon_s’.

**Optical Marker Label**	**Row**	**Optical Marker Label**	**Row**
L11	1:3	R31	31:33
L12	4:6	R32	34:36
R11	7:9	L41	37:39
R12	10:12	L42	40:42
L21	13:15	R41	43:45
L22	16:18	R42	46:48
R21	19:21	Hand m1	49:51
R22	22:24	Hand m2	52:54
L31	25:27	Belt m1	55:57
L32	28:30	Belt m2	58:60
