# Corrigendum: Multimodal chest surface motion data for respiratory and cardiovascular monitoring applications

**DOI:** 10.1038/sdata.2017.99

**Published:** 2017-08-01

**Authors:** Ghufran Shafiq, Kalyana Chakravarthy Veluvolu

**Keywords:** Radiotherapy, Diagnosis, Biomedical engineering

*Scientific Data* 4:170052 doi: 10.1038/sdata201752 (2017); Published 25 April 2017; Updated 1 August 2017

The financial support for this Data Descriptor was not properly acknowledged. The Acknowledgements section should have recognised a grant from the National Research Foundation of Korea (NRF), funded by the Ministry of Science, ICT & Future Planning of the Government of South Korea (grant no. 2017R1A2B2006032).

